# HEG1 is a novel mucin-like membrane protein that serves as a diagnostic and therapeutic target for malignant mesothelioma

**DOI:** 10.1038/srep45768

**Published:** 2017-03-31

**Authors:** Shoutaro Tsuji, Kota Washimi, Taihei Kageyama, Makiko Yamashita, Mitsuyo Yoshihara, Rieko Matsuura, Tomoyuki Yokose, Yoichi Kameda, Hiroyuki Hayashi, Takao Morohoshi, Yukio Tsuura, Toshikazu Yusa, Takashi Sato, Akira Togayachi, Hisashi Narimatsu, Toshinori Nagasaki, Kotaro Nakamoto, Yasuhiro Moriwaki, Hidemi Misawa, Kenzo Hiroshima, Yohei Miyagi, Kohzoh Imai

**Affiliations:** 1 Kanagawa Cancer Center Research Institute, Yokohama, Japan; 2 Department of Pathology, Kanagawa Cancer Center, Yokohama, Japan; 3 Department of Pathology, Kanagawa Cardiovascular and Respiratory Center, Yokohama, Japan; 4 Department of Pathology, Yokohama Municipal Citizen’s Hospital, Yokohama, Japan; 5 Division of General Thoracic Surgery, Yokosuka-Kyosai Hospital, Yokosuka, Japan; 6 Division of Pathology, Yokosuka-Kyosai Hospital, Yokosuka, Japan; 7 Department of General Thoracic Surgery and Asbestos Disease Center, Chiba Rosai Hospital, Ichihara, Japan; 8 Research Center for Medical Glycoscience, National Institute of Advanced Industrial Science and Technology, Tsukuba, Japan; 9 Division of Pharmacology, Faculty of Pharmacy, Keio University, Tokyo, Japan; 10 Department of Pathology, Tokyo Women’s Medical University, Yachiyo Medical Center, Yachiyo, Japan; 11 Institute of Medical Science, University of Tokyo, Tokyo, Japan

## Abstract

The absence of highly specific markers for malignant mesothelioma (MM) has served an obstacle for its diagnosis and development of molecular-targeting therapy against MM. Here, we show that a novel mucin-like membrane protein, sialylated protein HEG homolog 1 (HEG1), is a highly specific marker for MM. A monoclonal antibody against sialylated HEG1, SKM9-2, can detect even sarcomatoid and desmoplastic MM. The specificity and sensitivity of SKM9-2 to MM reached 99% and 92%, respectively; this antibody did not react with normal tissues. This accurate discrimination by SKM9-2 was due to the recognition of a sialylated *O*-linked glycan with HEG1 peptide. We also found that gene silencing of HEG1 significantly suppressed the survival and proliferation of mesothelioma cells; this result suggests that HEG1 may be a worthwhile target for function-inhibition drugs. Taken together, our results indicate that sialylated HEG1 may be useful as a diagnostic and therapeutic target for MM.

Malignant mesothelioma (MM) is a fatal tumor caused by past exposure to asbestos^1^. MM victims number ~3,000, 5,000, and 1,300 per year in the United States, Western Europe, and Japan, respectively^1^,^2^. Globally, ~125 million people have been exposed to asbestos at a workplace, and are at risk of developing MM^3^. Numerous Asian countries, particularly China, continue to use asbestos^4^. The 2011 Tohoku, Japan earthquake and tsunami destroyed over 100,000 houses^5^, probably scattering significant asbestos dust. This asbestos had been utilized in housing materials long ago. Present-day prohibition of domestic asbestos use is not sufficient to prevent asbestos exposure. Asbestos exposure continues to jeopardize public health.

The prognosis for MM patients is very poor. Most patients (77%) cannot receive cancer-directed surgery, and their median survival is ~7 months^6^. Satisfactory recovery is often not possible with chemotherapy and/or radiotherapy. The median survival of patients following these treatments is 9–12 months^6^,^7^. An effective and established molecular-targeting therapy against MM does not yet exist because a highly specific MM marker has not yet been discovered.

The absence of MM markers also influences diagnosis. MM differentiates into various histologic subtypes, and MM tumor antigens expressed specifically by each subtype have not been found^8^. Although some immunohistochemical positive markers currently used can detect MM at ~80% sensitivity, their specificities are insufficient^8^,^9^,^10^,^11^. These markers often have difficulty discriminating epithelioid MM from metastatic tumors or sarcomatoid MM from some sarcomas.

A mucin-like membrane protein, which is a membrane-anchored protein modified with many glycans, can be a good cancer-related antigen^12^. Immature glycans are produced by irregular processes of carbohydrate chain synthesis in tumor cells, and are attached in clusters on mucin-like proteins^12^,^13^,^14^. Some monoclonal antibodies (mAbs) recognizing irregular glycan clusters are used clinically to measure serologic tumor markers^12^,^15^. Moreover, the combined recognition of a mucin-like protein and its irregular glycan attachment can detect malignant tumor cells accurately^16^. A tumor-specific mucin-like membrane protein can also become a target for antibody-utilized immunotherapy^17^ or drug inhibition of cell proliferation^18^,^19^,^20^,^21^. If a mucin-like membrane protein with characteristic glycosylation is found on MM, it could become a specific target for accurate MM diagnosis or molecular-targeting therapy. However, changes in post-translational modifications are difficult to discover in cyclopedic analyses of gene expression. In addition, a large and heterogeneous molecular size, multiple charge states, and many heterogeneous glycans complicate detection of mucin-like proteins *via* general proteome analysis. Most mucin-like cancer antigens have been found by establishing tumor-specific mAbs. There are no shortcuts to find a tumor-specific mucin-like protein.

Protein HEG homolog 1 (HEG1) was first reported as the *heart of glass* gene regulating the concentric growth of the zebrafish heart^22^. The mouse HEG1 gene has been linked to cardiovascular organ development^23^. However, the function and structure of HEG1 has remained unclear. Here we show that sialylated HEG1, which we identified as a novel mucin-like membrane protein, is indeed a mesothelioma-related antigen, and that HEG1 expression supports the survival and proliferation of mesothelioma cells. Sialylated HEG1 may be a worthwhile target for MM diagnosis and therapy.

## Results

### Expression of SKM9-2 antigen in MM

**Figure 1:**
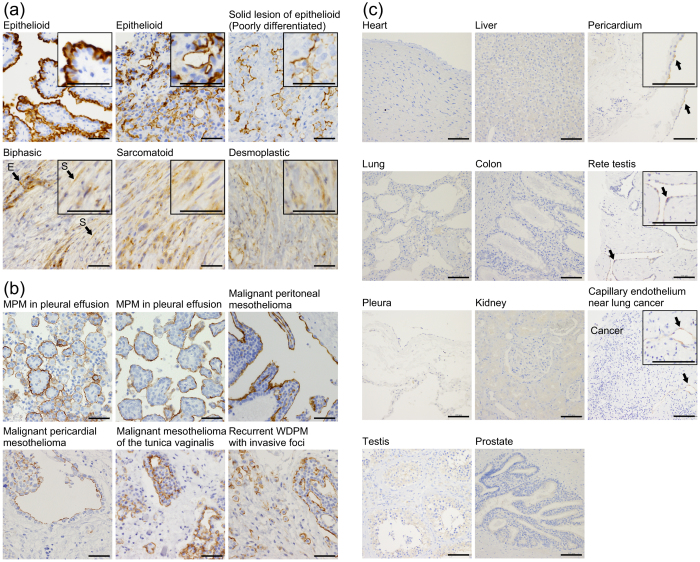
Representative images of immunohistochemical staining using mAb SKM9-2 in various histologic subtypes of MM or non-neoplastic tissues. (**a**) Immunostaining of various MPM samples. An enlarged image is shown on the upper right side. In biphasic MPM, epithelioid components or sarcomatoid components are indicated with the arrow of E or the arrow of S, respectively. Scale bars, 50 μm. (**b**) Immunostaining of rare mesothelioma and MPM cells in pleural effusion. Scale bars, 50 μm. (**c**) Immunostaining of non-neoplastic tissues. In positive cases, an enlarged image is shown on the upper right side. The positive cells are indicated by arrows. Capillary endothelium was partially stained in the angiogenesis around a tumor (arrow in the right bottom image). Scale bars, 100 μm.

The mAb SKM9-2 was obtained by immunizing epithelioid malignant pleural mesothelioma (MPM) cell lines to mice, and screening for mAb clones that recognized MPM cell lines but not a lung cancer cell line (Supplementary Methods and Supplementary Fig. 1). We investigated expression of SKM9-2 antigen in 130 cases of MPMs by immunohistochemistry (Table 1). SKM9-2 antigen was detected in 92% of MPMs, and the positive rate exceeded those for other MM diagnostic markers, *viz.* calretinin (80%), cytokeratin 5/6 (CK5/6) (78%), podoplanin (82%), nucleus Wilms’ tumor gene product 1 (WT-1) (87%), and mesothelin (79%). SKM9-2 antigen was effective in detecting sarcomatoid (64%) or desmoplastic MPMs (50%) that were insufficiently stained by other markers (0–50%). As shown in Fig. 1a, SKM9-2 antigen was mainly present on the apical cell membrane in epithelioid MPM and epithelioid components in biphasic MPM; in contrast, this antigen was weakly detected in the cytoplasm in sarcomatoid MPM, sarcomatoid components in biphasic MPM, or desmoplastic MPM. Such a shift in cellular localization has been reported on some membrane-associated mucins^20^,^24^,^25^,^26^,^27^. In solid lesions of epithelioid MPM, SKM9-2 antigen was negative or weakly positive in the cell membrane and/or the cytoplasm. The mAb SKM9-2 also stained the cytoplasmic membrane of MPM cells in pleural effusion (7/8, 88%) and rare cell types comprising malignant peritoneal mesotheliomas (3/3, 100%), a malignant pericardial mesothelioma, a malignant mesothelioma of the tunica vaginalis, and a recurrent well-differentiated papillary mesothelioma with invasive foci in the peritoneum (Fig. 1b). These results suggest that the SKM9-2 antigen is a sensitive histopathological marker for various MM types.

### High specificity of SKM9-2 antigen to MM; insignificant expression of SKM9-2 antigen in non-mesothelioma tumors and non-neoplastic tissues

We investigated the expression of SKM9-2 antigen on non-mesothelioma tumors by using tissue microarrays of 24 primary tumors. SKM9-2 antigen was expressed in only 3/310 cases of unrelated tumors (Supplementary Fig. 2). The MM specificity of SKM9-2 antigen reached 99%, which was the highest specificity that we measured among MM markers (Table 2). Although nuclear WT-1 is a good MM marker, WT-1 protein was also expressed in the cytoplasm of non-tumor cells and other tumors (Supplementary Fig. 3). Because of the cytoplasmic staining for WT-1, it was often difficult to clearly discern nuclear staining for WT-1 (Supplementary Fig. 3, bottom panel). Relative to WT-1, the SKM9-2 antigen exhibited better visibility.

SKM9-2 antigen was not detected in major organs (Fig. 1c). The low expression of SKM9-2 antigen by normal tissues is summarized in Supplementary Table 1. A few positive cases, *viz.* mesothelial cells of the pericardium, epithelium of the rete testis, and capillary endothelium, are shown in Fig. 1c right panels (arrow). The mAb SKM9-2 often stained the capillary endothelium in near-tumor angiogenesis. This mAb was also reactive on some activated mesothelial cells, especially from a pneumothorax case. In six cases of fibrous pleurisy, spindle cells were not stained with mAb SKM9-2, whereas reactive mesothelial cells in two cases were stained with the antibody. These results suggest that SKM9-2 antigen has low expression in non-mesothelioma cells and normal tissues, except in a part of the capillary endothelium, and reactive mesothelial cells.

In summary, the mAb SKM9-2 can detect MM with 99% specificity and 92% sensitivity on histopathological specimens, which is better performance than that provided by other major MM diagnostic markers. The mAb SKM9-2 may be a good MM-specific marker for pathological diagnosis.

### mAb SKM9-2 recognizes a sialylated HEG1

**Figure 2:**
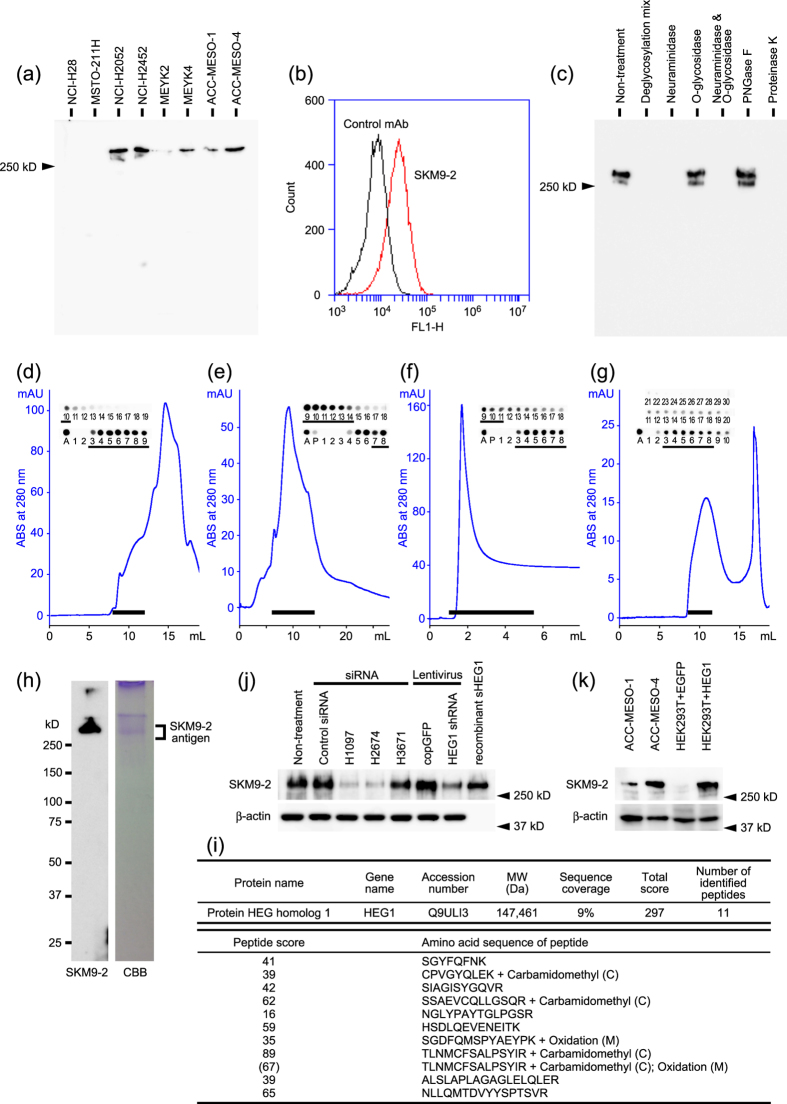
mAb SKM9-2 recognizes a sialylated HEG1 on the cell surfaces of mesothelioma cells. (**a**) Representative immunoblotting (n = 2) of SKM9-2 antigen in MPM cell lines. Cell lysates (10 μL per lane) were resolved by SDS-PAGE under non-reducing conditions. (**b**) Flow cytometric analysis of SKM9-2 antigen on ACC-MESO-4. An irrelevant mAb (2D2, mouse IgG1) was used as a negative control mAb. (**c**) Elimination of recognition with mAb SKM9-2 by glycosidase treatment. A partially purified sample was treated with glycosidase or proteinase K and resolved by SDS-PAGE under reducing conditions. Similar results were obtained by 3 independent analyses. PNGase F, peptide N-glycosidase F. (**d**,**e**,**f**,**g**) Purification of SKM9-2 antigen using a Superose 6 Increase 10/300GL, Mono Q 5/50GL, WGA-agarose, or Superose 6 Increase 10/300GL column, respectively. Dot blot results are also shown. Pooled fractions are indicated as a black bar. (**h**) Western blotting and CBB staining of purified SKM9-2 antigen. (**i**) Results of Mascot search for purified SKM9-2 antigen. The purified sample was resolved by SDS-PAGE. The CBB-stained band was then cut from the gel and analyzed by mass spectrometry and Mascot search. The highest-scoring hit is shown. (**j**) Decrease of SKM9-2 antigen by gene silencing of HEG1. Cell lysates (7.5 μL per lane) were resolved by SDS-PAGE under reducing conditions and detected by western blotting using mAb SKM9-2. Similar results were obtained by 3 independent analyses. After detection, the membrane was reprobed and treated with anti-β-actin mAb (AC-15). Control siRNA, MISSION siRNA Universal Negative Control (SIC-001); H1097, H2674, and H3671, HEG1 siRNAs; copGFP, a control lentiviral particles. (**k**) Representative western blotting (n = 2) of HEG1-transfected HEK293T (HEK293T + HEG1) using mAb SKM9-2. Cell lysates (5 μL per lane) were resolved by SDS-PAGE under reducing conditions. EGFP-transfected HEK293T (HEK293T + EGFP) was used as a negative control. After detection, the membrane was reprobed and treated with anti-β-actin mAb (AC-15).

SKM9-2 antigen was detected in lysates of several MPM cell lines as ~400 kDa bands on western blots (Fig. 2a). Flow cytometric analysis (Fig. 2b) suggested that SKM9-2 antigen was expressed on the cell surface. Neuraminidase treatment eliminated recognition of mAb SKM9-2, whereas treatment with peptide N-glycosidase F or O-glycosidase alone, which cannot digest sialylated *O*-linked glycans, did not affect the reactivity of mAb SKM9-2 (Fig. 2c). Proteinase K treatment also eliminated binding of mAb SKM9-2 (Fig. 2c). We reason that the mAb SKM9-2 would recognize a sialylated *O*-linked glycan in conjunction with peptide sequences of a large mucin-like membrane protein.

SKM9-2 antigen was purified from ACC-MESO-4, a mesothelioma cell line, by precipitation under acidic conditions and column chromatography (see Supplementary Methods). SKM9-2 antigen was fractionated as a very large molecule near the void volume on size-exclusion chromatography (Fig. 2d and g), eluted as a broader peak in anion exchange chromatography (Fig. 2e), and purified by WGA-agarose to which sialomucin bound (Fig. 2f). These biochemical properties suggest that SKM9-2 antigen is a heavily sialylated mucin.

A 400 kDa SDS-PAGE band stained with Coomassie brilliant blue (CBB) was concordant with the band detected by western blotting using mAb SKM9-2 (Fig. 2h). The band was cut from CBB-stained gel, and analyzed using nano-LC MS/MS of trypsinized peptides and a subsequent Mascot search. Results of this analysis suggest that SKM9-2 antigen is HEG1 (Fig. 2i).

To confirm that SKM9-2 antigen is sialylated HEG1, we performed a gene silencing analysis of HEG1. Suppressing the HEG1 gene with 3 different regions of siRNA (H1097, H2674, and H3671) specifically decreased HEG1 western blot signals (Fig. 2j). Lentiviral particles coding HEG1 shRNA also lightened the band detected with mAb SKM9-2, in contrast to the control lentiviral particles (copGFP). Recombinant soluble HEG1 (sHEG1) in which a His-tag was substituted for the transmembrane and cytoplasmic domains was purified from the culture supernatant of sHEG1-transfected ACC-MESO-4. The mAb SKM9-2 recognized sHEG1 at a molecular size similar to that of native HEG1 (Fig. 2j). In addition, the mAb SKM9-2 bound to a molecule in HEG1-transfected HEK293T, but not EGFP-transfected cells (Fig. 2k).

### Genomic structure of human HEG1

**Figure 3:**
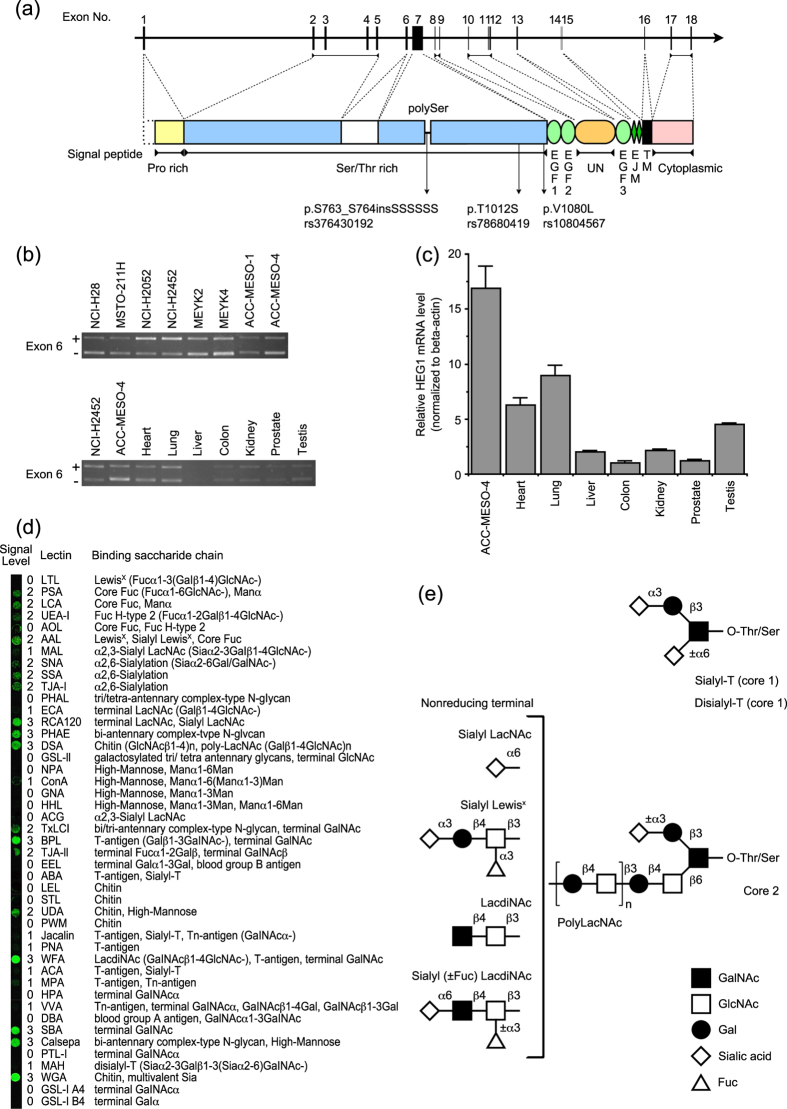
Structure and expression of human HEG1. (**a**) Structure of open reading frame of human HEG1 gene. HEG1 gene sequences were retrieved from GenBank (accession no. NC_000003, region 124965710–125055958). The positions of SNPs in HEG1 of ACC-MESO-4 are indicated with the reference SNP ID number at the bottom. The domain structure of HEG1 was predicted as follows: signal peptides (exon 1), residues 1–29; proline rich domain (Pro rich) (exon 1), 30–106; serine/threonine rich region (Ser/Thr rich) (exon 2–5), 107–530; Ser/Thr rich (exon 6), 531–630; Ser/Thr rich (exon 7), 631–1086, including poly-serine sequences (polySer), 759–772; EGF domain 1 (typical EGF motif) (exon 8), 1087–1125; EGF domain 2 (Ca^2+^-binding EGF motif) (exon 9), 1126–1165; unknown region (UN) (this domain may contain a potential proteolytic cleavage site) (exon 10–12), 1166–1273; EGF domain 3 (this domain may form a laminin-type EGF-like domain with a following extracellular juxtamembrane region (EJM) 1) (exon 13), 1274–1318; EJM 1 (EJMs consist of a short loop formed by a disulfide-bridge) (exon 14), 1319–1332; EJM 2 (exon 15), 1333–1345; transmembrane domain (TM) (exon 16), 1346–1370; cytoplasmic domain (exon 16–18), 1371–1481. (**b**) Representative image of RT-PCR (n = 2) of HEG1 with or without exon6. PCR products were resolved at the length of 600 bp or 900 bp. (**c**) Real-time quantitative PCR of HEG1 in human organs. Values were normalized to the amount of β-actin, and are reported as means ± S.D. of tetraplicate determinations. Similar results were obtained in 2 independent experiments. (**d**) Glycosylation analysis of HEG1. Purified HEG1 (Fig. 2h) was used as a sample. The signal level was estimated by subtracting the fluorescence intensity of the control without antigen from that of the sample containing SKM9–2 antigen. Original images are shown in Supplementary Fig. 5. (**e**) Predicted structure of *O*-glycan attaching to HEG1.

The structure and function of full-length human HEG1 have not yet been investigated. Figure 3a shows the genomic structure of human HEG1. The human HEG1 gene (*HEG1*) is located between mucin-13 (*MUC13*) and solute carrier family 12 member 8 (*SLC12A8*) in chromosome 3q21.2 (GeneCards human gene database). Human HEG1 is predicted to be a type I membrane protein and to have a longer Ser/Thr rich region than the HEG1 reported for mice and zebrafish (Fig. 3a and Supplementary Fig. 4). This Ser/Thr rich region accounts for ~70% of the mature HEG1 peptide, and is followed by three epidermal growth factor (EGF)-like domains, an unknown region, two extracellular juxtamembrane regions, a transmembrane domain, and a cytoplasmic domain (Fig. 3a). HEG1 would contain many *O*-linked glycans in the Ser/Thr rich region; because the molecular size of purified HEG1 was 400 kDa, which is much larger than its predicted size of 150 kDa (Fig. 2a); HEG1 showed mucin-like characteristics during purification, as mentioned above (Fig. 2d–g); and its various *O*-linked glycans were detected in a lectin microarray analysis described later (Fig. 3d–e). HEG1 does not belong to the mucin gene family because it does not contain typical tandem repeat structures. However, HEG1 has a long *O*-glycosylated region and EGF domains, such as membrane-associated mucins^12^. HEG1 may have a physiological function similar to that of a membrane-associated mucin.

Two transcript variants of HEG1 with or without exon 6 were cloned from ACC-MESO-4. In an RT-PCR analysis, mRNAs of both variants were transcribed without a distinct bias in all tested MPM cell lines (Fig. 3b, upper panel). Thus, the two variants of HEG1 would be expressed on the cells concurrently. Slightly longer PCR products of ACC-MESO-1 or ACC-MESO-4 (Fig. 3b, upper panel), but not other cells, resulted from insertion of an additional 6 Ser residues in a characteristic poly-Ser region (the SNP present in dbSNP137) (Fig. 3a). HEG1 of ACC-MESO-4 also had two missense SNPs (Fig. 3a). Binding of mAb SKM9-2 was not affected by the presence or absence of exon 6 or these SNPs in ACC-MESO-4.

### Glycosylation of HEG1 in mesothelioma cells

Glycosylation of HEG1 was investigated using a lectin microarray^28^ (Fig. 3d and Supplementary Fig. 5). Abbreviations of lectins are shown in the Methods section. HEG1 bound to AAL but not LTL, which indicates that HEG1 has sialyl Lewis^X^, but not Lewis^X^. HEG1 also bound to other Fuc binders (PSA, LCA, UEA-I). HEG1 characteristically reacted with α2,6-linked sialic acid binders (SNA, SSA, TJA-I) and a sialylated *N*-acetyllactosamine (LacNAc) binder (RCA120), but not to a terminal LacNAc binder (ECA) or α2,3-sialyl LacNAc binders (MAL, ACG). These observations suggest that α2,6-sialyl LacNAc is attached to the nonreducing terminal of HEG1 glycan. The signals for binders of T-antigen with or without sialic acid (ABA, jacalin, PNA, ACA, and MPA) and a disialyl T-antigen binder (MAH) were weak or undetectable. Although sialyl T-antigen or disialyl T-antigen was attached to HEG1, these antigens may be present at comparatively low levels, or they may be screened by large glycans. The binding of HEG1, but not chitin binders (LEL, STL, PWN), to WGA and DSA suggests that HEG1 is a sialomucin containing a poly-LacNAc structure. The strong reaction of HEG1 to terminal GalNAc binders (TxLCI, BPL, TJA-II, and SBA), but not α-GalNAc binders (HPA, DBA, PTL-I, and GSL-I A4), indicates that some terminals of HEG1 glycan contain β-GalNAc. Furthermore, the binding of HEG1 to WFA suggests that HEG1 has a GalNAcβ1-4GlcNAc [*N, N’*-diacetyllactosamine (LacdiNAc)] structure. Considering these results, the structure of *O*-glycan attached to HEG1 is summarized in Fig. 3e. HEG1 is attached with sialyl T-antigen, disialyl T-antigen, and many core 2 glycans with a poly-LacNAc structure. The nonreducing terminal of this poly-LacNAc structure would be attached with α2,6-linked sialic acid, sialyl Lewis^x^, or LacdiNAc with or without α2,6-linked sialic acid and/or α1,3-linked fucose.

Although SKM9-2 antigen was not detected in major organs (Fig. 1c), HEG1 mRNA was observed in the heart and the lung (Fig. 3c and b, lower panel). HEG1 mRNA expression in the liver, colon, kidney, prostate, and testis was relatively low (Fig. 3c). Although HEG1 protein may have low expression in some tissues other than MM, the mAb SKM9-2 can distinguish only HEG1 with glycosylation characteristic of MM.

### HEG1-dependent proliferation of mesothelioma cells

**Figure 4:**
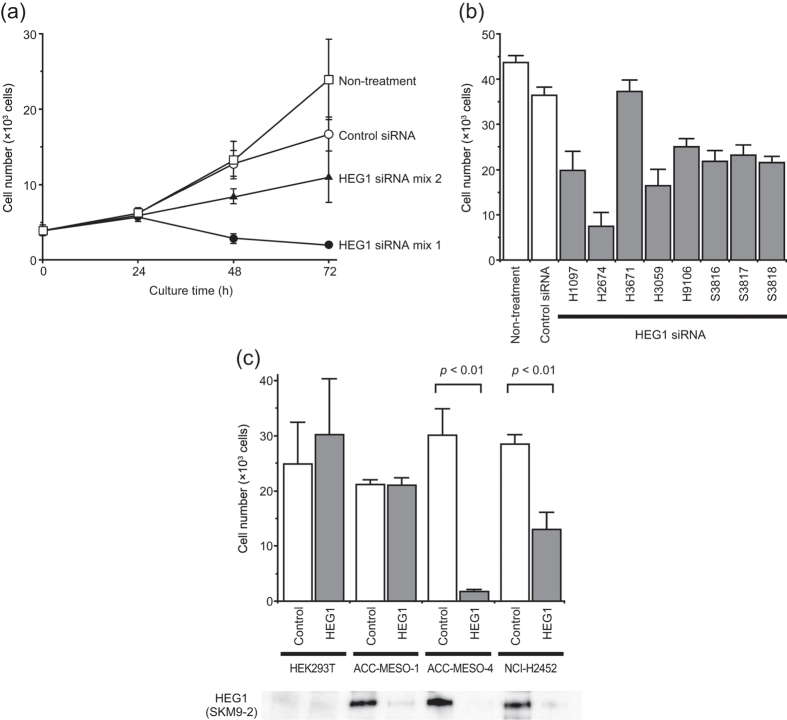
Suppression of proliferation of mesothelioma cells by HEG1 siRNA. Values are means ± S.D. of tetraplicate determinations. (**a**) Time-course of cell growth of ACC-MESO-4 treated with HEG1 siRNA. siRNA was transfected with Lipofectamine 2000. HEG1 siRNA mix 1, a mixture of H1097 and H2674 (1:1); HEG1 siRNA mix 2, sc-78365 (Santa Cruz Biotechnology); Control siRNA, MISSION siRNA Universal Negative Control (SIC-001). Similar results were obtained in 2 independent experiments. (**b**) Suppression of cell proliferation of ACC-MESO-4 with several HEG1 siRNAs. The cells were cultured for 72 h with siRNA and Lipofectamine 2000. Control siRNA, MISSION siRNA Universal Negative Control. Similar results were obtained in 2 independent experiments. (**c**) Suppression of cell proliferation with HEG1 siRNA on MPM cell lines. The cells were treated for 72 h with siRNA and Lipofectamine RNAiMAX. HEG1 expression after 72 h was shown in western blotting using mAb SKM9-2 at the bottom of the figure. Differences between mean values were analyzed by Student’s *t*-test. *P*-values for tests of ACC-MESO-4 and NCI-H2452 were <0.01. Differences for other cells were not significant. Control was treated with a control siRNA, MISSION siRNA Universal Negative Control; HEG1 was treated with a HEG1 siRNA, H2674. Similar results were obtained in 2 independent experiments.

Sialylated HEG1 was expressed on proliferative cells such as reactive mesothelial cells, endothelial cells in angiogenesis, and mesothelioma (Fig. 1). Since the membrane-associated mucins MUC1, MUC4, MUC13, and MUC16 support the survival and proliferation of cancer cells^18^,^19^,^20^,^21^, we expected that HEG1 would associate with cell proliferation. Proliferation of mesothelioma cells was suppressed by HEG1 siRNA, but not control siRNA, in a time-dependent manner (Fig. 4a). Inhibition of proliferation by HEG1 siRNA was also observed on the reduced incorporation of bromodeoxyuridine (Supplementary Fig. 6). Cell death was partially induced by HEG1 siRNA mix 1 (H1097 plus H2674) after 48 h of treatment. Some siRNAs diminished the cell growth by ~50%, and one siRNA (H2674) strongly inhibited cell proliferation (Fig. 4b). Control siRNA and H3671 (a weak silencer of HEG1, Fig. 2j) did not significantly affect cell growth (Fig. 4b). The suppression of cell growth by H2674 was also observed on NCI-H2452, another mesothelioma cell line (Fig. 4c). In contrast, the cell growth rates of HEK293T and ACC-MESO-1, which expressed lower amounts of SKM9-2 antigen (Fig. 2k), were not affected by the siRNA (Fig. 4c). These results suggest that MM cell proliferation partly depends on HEG1 expression. The more HEG1 is expressed on the cells, the more dependently the cells may proliferate.

## Discussion

Antibody against a specific glycosylation site on a membrane protein can exhibit high specificity to tumor cells^16^. This high specificity makes it possible to provide chimeric antigen receptor therapy that is more potent than tumor vaccine or antibody therapy^17^. The mAb SKM9-2 recognizes both HEG1 peptide and its sialylated *O*-glycosylation, and binds to MM with high specificity and sensitivity. This dual recognition of mAb SKM9-2 would be advantageous to a molecular-targeting therapy against MM using antibody. Furthermore, we found that HEG1 on mesothelioma cells contains a unique LacdiNAc structure. LacdiNAc is a rare glycosylation in human proteins, previously observed in gastric mucin and cancer antigens^29^,^30^,^31^,^32^. We expect that a more accurate diagnosis of MM will become possible by combining detection of SKM9-2 antigen with that of the LacdiNAc modification on HEG1.

We showed that sialylated human HEG1 was mainly expressed on the apical membrane, unlike the reported mouse HEG1 observed in the cell-cell junction^23^. Sialylated human HEG1 expression on the apical surface is consistent with this protein’s heavy glycosylation and hydrophilicity. Mesothelioma cells disseminated into pleural effusion also expressed sialylated HEG1 on the cell cluster apical surface, but not at cell-cell junctions (Fig. 1b). This HEG1 expression seemed to prevent re-attachment of mesothelioma cells detached from the pleura as well as anti-adhesion effects of membrane-associated mucin^12^,^20^,^33^.

Membrane-associated mucin acts as a physical barrier and performs various physiological functions related to cell survival^12^,^34^. The EGF domain of MUC4 binds to ErbB2 (HER2/neu)^19^. An EGF domain of HEG1, instead of MUC4, may associate with ErbBs as a proliferation-regulating mucinous molecule on the mesothelioma cells, because MUC4 is not expressed on mesothelioma^35^. An EGF domain released from HEG1 may also bind to EGFR, analogous to binding reported for other EGFR ligands^36^,^37^. Although a proteolytic cleavage site characteristic of membrane-associated mucins was not found in HEG1, we speculate that the unknown region conserved among mammals (Fig. 3a and Supplementary Fig. 4) is a cleavage site digested by proteases, and that the released EGF domain of HEG1 functions as a growth factor. The cytoplasmic domain of MUC1 interacts with receptor tyrosine kinases and PI3Ks^18^. The cytoplasmic domain in HEG1 contains a predicted phosphorylation site (Ser1393) and is bound by KRIT1, an intracellular molecule^38^. HEG1 does not have a typical PxxP motif for binding protein kinase; however, HEG1 does have a P/Y/R/K rich region (1394-PYAEYPKNPR-1403), which may be bound with Src homology 3 domain^39^. We expect that the HEG1 cytoplasmic domain associates with signaling molecules for cell proliferation. Functional inhibitors of HEG1 may have anti-mesothelioma activity; a MUC1 inhibitor has been examined for anti-cancer properties^34^.

For efficacious cancer immunotherapy using antibodies, vaccines, or chimeric antigen receptors against a tumor antigen, it is desirable to target a molecule expressed specifically on the tumor. Mucin-like membrane proteins may be worthwhile targets for cancer immunotherapy due to their tumor-specific patterns of glycosylation^40^,^41^. In light of the association between HEG1 and mesothelioma proliferation, the mAb SKM9-2 and HEG1 may be productive diagnostic tools and therapeutic targets for MM.

## Methods

Descriptions of screening of mAb against MPM, western blotting, flow cytometric analysis, deglycosylation analysis, purification of SKM9-2 antigen, cloning of HEG1 and production of recombinant HEG1, RT-PCR, and real-time quantitative PCR are described in the Supplementary Methods.

### Cell lines

Human MPM cell lines: ACC-MESO-1 (RCB2292) and ACC-MESO-4 (RCB2293)^42^ were obtained from RIKEN Cell Bank (Tsukuba, Japan); NCI-H28 (CRL-5820), NCI-H2052 (CRL-5915), NCI-H2452 (CRL-5946), and MSTO-211H (CRL-2081) were obtained from ATCC (Manassas, VA, USA); and MEYK2 and MEYK4 were established from cells in pleural effusions of Japanese MPM patients^43^. Human embryonic kidney cell line HEK293T (RCB2202) and human lung adenocarcinoma cell line A549 (RCB0098) were obtained from RIKEN Cell Bank (Tsukuba, Japan). Mouse myeloma cell line PAI (JCRB0113) was obtained from JCRB Cell Bank (Osaka, Japan).

### Tissues

The study was approved by the ethics committees of Kanagawa Cancer Center. Informed consent was obtained from all patients or their relatives. All experimental protocols were approved by Kanagawa Cancer Center. All experiments were performed in accordance with relevant guidelines and regulations. We obtained tissue samples of the cholecyst and breast from patients surgically treated at Kanagawa Cancer Center between 2006 and 2010. Other non-tumorous tissue samples were obtained from patient specimens anatomized within 4 h following death at Kanagawa Cancer Center between 2006 and 2010. The samples of pleurisy (6 cases) and pneumothorax (1 case), which were used for analysis of SKM9-2 binding to reactive mesothelioal cells and/or spindle cell proliferation, were obtained by video-assisted thoracoscopic surgery at Chiba-Rosai Hospital between 2010 and 2015. Mesothelioma samples were obtained from patients who were diagnosed with malignant mesothelioma at Kanagawa Cancer Center, Yokohama Municipal Citizen’s Hospital, Kanagawa Cardiovascular and Respiratory Center, Yokosuka-Kyosai Hospital, Chiba-Rosai Hospital, or Tokyo Women’s Medical University Yachiyo Medical Center between 1997 and 2016. Other tumor tissues were obtained from primary tumors of patients who were pathologically diagnosed as having tumors at Kanagawa Cancer Center between 1999 and 2012. MPM cells in pleural effusion were provided as formalin-fixed paraffin-embedded cell blocks. All specimens were prepared as formalin-fixed paraffin-embedded thin-sliced sections. The specimens, except those for mesothelioma, urothelial carcinoma, and pleurisy, were used as tissue microarrays, with the tissue samples cut to a circle 1 or 3 mm in diameter. The authors K.H. and K.W. reviewed all of the cases of mesotheliomas and pleurisy, and confirmed the histopathlogical diagnosis.

### Immunohistochemistry

The sections on glass slides were treated by the autostainer according to the manufacturer’s instructions; calretinin, mesothelin, or a part of SKM9-2 antigen (identified as sialylated HEG1 in this study) was used with Histostainer 48 A (Nichirei Co., Tokyo, Japan); CK5/6, podoplanin, or WT-1 was used with Ventana BenchMark ULTRA (Ventana Medical Systems, Tucson, AZ). The antigen retrieval protocol was as follows: calretinin was heated for 40 min at 98 °C in Heat Processor Solution pH 9 (Nichirei Co.); mesothelin or SKM9-2 antigen was heated at 98 °C for 40 min or 121 °C for 10 min, respectively, in Target Retrieval Solution, Citrate pH 6 (Dako Japan Co, Kyoto, Japan); cytokeratin 5/6 (CK5/6) or podoplanin was heated at 95 °C for 64 min in Ultra Cell Conditioning Solution (ULTRA CC1) (Ventana Medical Systems); Wilms’ tumor gene product 1 (WT-1) was heated at 95 °C for 64 min in Ultra Cell Conditioning Solution (ULTRA CC2) (Ventana Medical Systems). The primary antibodies used were as follows: calretinin, rabbit anti-calretinin polyclonal antibody (PAD:DC8) (Thermo Fisher Scientific, Rockford, IL, USA); mesothelin, mouse anti-human mesothelin mAb (5B2) (Leica Microsystems, Bannockburn, IL); CK5/6, mouse anti-cytokeratin 5,6 mAb (D5/16B4) (Nichirei Co.); podoplanin, mouse anti-podoplanin mAb (D2-40) (Ventana Medical Systems); and WT-1, mouse anti-human WT-1 mAb (6F-H2) (Ventana Medical Systems). Each antibody was used according to the respective manufacturer’s instructions. SKM9-2 antigen was stained with the culture supernatant of a hybridoma clone (SKM9-2) containing mouse anti-human HEG1 mAb. Immunoreactivity was visualized using Histofine Simple Stain MAX-PO (Multi) (Nichirei Co.) with Liquid DAB+ (Dako Japan Co) [calretinin and mesothelin], the EnVision+ kits [SKM9-2 antigen], or the ultraView Universal DAB Detection Kit (Ventana Medical Systems) [CK5/6, podoplanin, and WT-1], according to the manufacturer’s instructions. Finally, the sections were counterstained with hematoxylin, dehydrated, and mounted with Malinol medium (Muto Pure Chemicals, Tokyo, Japan). Immunostaining was evaluated on the basis of the intensity and proportion of staining on all tumor cells in each specimen. To evaluate the immunostaining for calretinin or WT-1, the staining in the nucleus, but not the cytoplasm, was measured. The intensity of staining was defined by scoring as follows: 2, strong staining; 1, weak staining; 0, no staining. The proportion of staining was measured in the entire microscopic field of tumor cells for each specimen and was classified by scoring as follows: 3, >50%; 2, 50–10%; 1, 9-1%; 0, 0%. Cases were defined as positive if the proportion score was more than 0.

### Gene silencing

For siRNA gene silencing, ACC-MESO-4 (70% confluent in a 24-well plate) was cultured for 72 h in 600 μL of culture medium with 100 pmol of siRNA and 1 μL of Lipofectamine 2000 (Thermo Fisher Scientific). The gene silencing with shRNA was performed using commercially available lentiviral particles (Santa Cruz Biotechnology, Dallas, TX, USA). ACC-MESO-4 (70% confluent in a 24-well plate) was treated for 48 h with 5 × 10^4^ infectious units of lentiviral particles according to the manufacturer’s instructions, and cells stably expressing shRNA were selected for 10 days by culturing with 10 μg/mL puromycin. These cells were solubilized and analyzed by western blotting as described in Supplementary Methods. siRNA against human HEG1 was designed by Enhanced siDirect (http://rnai.co.jp/lsci/license.html); H1097, 5′-GAUCUUUGACGGUCAGUCUGG-3′ and 5′-AGACUGACCGUCAAAGAUCGC-3′; H2674, 5′-CCUAUAGCCGUACAGACUACA-3′ and 5′-UAGUCUGUACGGCUAUAGGGC-3′; H3671, 5′-GCAAGUCGGGAUACUUUCAGU-3′ and 5′-UGAAAGUAUCCCGACUUGCAC-3′. As a negative control for the siRNA assay, MISSION siRNA Universal Negative Control (SIC-001) (Sigma-Aldrich Japan K.K., Tokyo, Japan) was used. Human HEG1 shRNA lentiviral particles (sc-78365-V) and copGFP control lentiviral particles (sc-108084) were obtained from Santa Cruz Biotechnology.

### Lectin microarray

Purified HEG1 (Fig. 2h) was used as a sample. The sample (1.65 μg, 33 μL) was added to 47 μL of 50 mM Tris-buffered saline (pH 8.0) containing 1% Triton X-100 (TBSTx), and applied to an array of 45 lectins spotted in triplicate on a glass slide (LecChip ver. 1.0; GlycoTechnica, Yokohama, Japan). After incubation at 20 °C for 18 h, the glass slide was washed with TBSTx, incubated with mAb SKM9-2 at 20 °C for 3 h, washed again with TBSTx, and treated with Cy3-conjugated donkey anti-mouse IgG (Jackson ImmunoResearch, West Grove, PA, USA) at 20 °C for 3 h. After washing, the slide was scanned using a GlycoStation Reader 1200 (GlycoTechnica). Lectins are abbreviated as follows: LTL, *Lotus tetragonolobus* lectin; PSA, *Pisum sativum* agglutinin; LCA, *Lens culinaris* agglutinin; UEA, *Ulex europaeus* agglutinin; AOL, *Aspergillus oryzae* lectin; AAL, *Aleuria aurantia* lectin; MAL, *Maackia amurensis* lectin; SNA, *Sambucus nigra* agglutinin; SSA, *Sambucus sieboldiana* agglutinin; TJA, *Trichosanthes japonica* agglutinin; PHAL, *Phaseolus vulgaris* leucoagglutinin; ECA, *Erythrina cristagalli* agglutinin; RCA, *Ricinus communis* agglutinin; PHAE, *Phaseolus vulgaris* erythroagglutinin; DSA, *Datura stramonium* agglutinin; GSL, *Griffonia simplicifolia* lectin; NPA, *Narcissus pseudonarcissus* agglutinin; ConA, concanavalin A; GNA, *Galanthus nivalis* agglutinin; HHL, *Hippeastrum hybrid* lectin; ACG, *Agrocybe cylindracea* galectin; TxLCI, *Tulipa gesneriana* lectin; BPL, *Bauhinia purpurea alba* lectin; EEL, *Euonymus europaeus* lectin; ABA, *Agaricus bisporus* agglutinin; LEL, *Lycopersicon esculentum* lectin; STL, *Solanum tuberosum* lectin; UDA, *Urtica dioica* agglutinin; PWM, Pokeweed mitogen; PNA, Peanut agglutinin; WFA, *Wisteria floribunda* agglutinin; ACA, *Amaranthus caudatus* agglutinin; MPA, *Maclura pomifera* agglutinin; HPA, *Helix pomatia* agglutinin; VVA, *Vicia villosa* agglutinin; DBA, *Dolichos biflorus* agglutinin; SBA, Soybean agglutinin; PTL, *Psophocarpus tetragonolobus* lectin; MAH, *Maackia amurensis* hemagglutinin; WGA, Wheat germ agglutinin; GSL, *Griffonia simplicifolia* lectin.

### Measurement of cell growth

Cell lines were seeded in a 96-well plate (5 × 10^3^ cells/well) and cultured for 24 h in 100 μL of culture medium. For transfection, 15 μl of Opti-MEM (Thermo Fisher Scientific), containing 7.5 pmol of siRNA and 0.15 μL of Lipofectamine 2000 or Lipofectamine RNAiMAX (Thermo Fisher Scientific), was added to the cells in each well and cultured for 24, 48, or 72 h. The viable cell count was measured by CellTiter 96 AQueous One Solution Cell Proliferation Reagent (Promega K.K., Tokyo, Japan). Several human HEG1 siRNAs designed by Enhanced siDirect were used; HEG1 siRNA mix 1, a mixture of H1097 and H2674 (1:1); H3059, 5′-GCGAAUGCGUCGCAGACAACA-3′ and 5′-UUGUCUGCGACGCAUUCGCCA-3′; and H9106, 5′-CUGGCGUUCUAGUCAGUAAAA-3′ and 5′-UUACUGACUAGAACGCCAGAC-3′. Four commercially available siRNAs against human HEG1 were also tested: HEG1 siRNA mix 2, sc-78365 (Santa Cruz Biotechnology); S3816, SASI_Hs02_00353816; S3817, SASI_Hs02_00353817; and S3818, SASI_Hs02_00353818 (Sigma-Aldrich Japan K.K.). As a negative control, MISSION siRNA Universal Negative Control (SIC-001) was used. HiLyte Fluor 488-labeled universal negative control siRNA was obtained from Nippon Gene Co (Tokyo, Japan). In tested cell lines, sufficient HiLyte Fluor-labeled siRNA was transfected with the lipofection reagent.

## Additional Information

**How to cite this article:** Tsuji, S. *et al*. HEG1 is a novel mucin-like membrane protein that serves as a diagnostic and therapeutic target for malignant mesothelioma. *Sci. Rep.* **7**, 45768; doi: 10.1038/srep45768 (2017).

**Publisher's note:** Springer Nature remains neutral with regard to jurisdictional claims in published maps and institutional affiliations.

## Supplementary Material

Supplementary Information

## Figures and Tables

**Table 1:** Expression rates of marker antigens in MPM.

MPM type	SKM9-2 antigen	Calretinin	CK5/6	Podoplanin	WT-1	Mesothelin
Epithelioid	89/91 (98%)	62/71 (88%)	60/71 (85%)	63/71 (89%)	64/71 (90%)	63/71 (89%)
Biphasic	19/21 (90%)	13/14 (93%)	12/14 (86%)	11/14 (79%)	12/14 (86%)	11/14 (79%)
Sarcomatoid	9/14 (64%)	1/8 (13%)	2/8 (25%)	3/8 (38%)	6/8 (75%)	1/8 (13%)
Desmoplastic	2/4 (50%)	0/2 (0%)	0/2 (0%)	1/2 (50%)	1/2 (50%)	0/2 (0%)
Total	119/130	76/95	74/95	78/95	83/95	75/95
Sensitivity	92%	80%	78%	82%	87%	79%

The histologic type of MPM was classified by pathological diagnosis. Intensity and proportion of staining of MPM cells were evaluated in the entire microscopic field of the specimen. Cases were defined as positive if the proportion score was more than 0. In the immunostaining of calretinin or WT-1, staining in the nucleus, but not the cytoplasm, was considered a positive sample.

**Table 2:** Expression rates of mesothelioma markers in non-MPM tumors.

	SKM9-2 antigen	Calretinin	CK5/6	Podoplanin	WT-1	Mesothelin
Lung carcinoma	0/98	23/98	41/98	13/98	0/98	44/98
Renal cell carcinoma	0/10	0/10	1/10	0/10	0/10	0/10
Gastric adenocarcinoma	0/10	2/10	7/10	6/10	0/10	1/10
Colon adenocarcinoma	0/10	0/10	0/10	0/10	0/10	0/10
Breast cancer	0/10	1/10	2/10	0/10	1/10	0/10
Ovary adenocarcinoma	0/10	0/10	7/10	1/10	3/10	6/10
Urothelial carcinoma	1/10	2/10	7/10	0/10	0/10	0/10
Carcinosarcoma	0/10	3/10	6/10	2/10	2/10	3/10
Liposarcoma	0/10	6/10	1/10	1/10	1/10	0/10
Rhabdomyosarcoma	0/10	1/10	0/10	2/10	1/10	0/10
Leiomyosarcoma	1/10	1/10	0/10	2/10	0/10	0/10
Fibrosarcoma	0/10	2/10	0/10	0/10	1/10	0/10
Synovial sarcoma	0/10	2/10	2/10	5/10	1/10	2/10
Gastrointestinal stromal tumor (GIST)	0/9	0/9	0/9	1/9	1/9	0/9
Ewing’s sarcoma	0/10	1/10	0/10	2/10	0/10	0/10
Alveolar soft part sarcoma (ASPS)	0/10	0/10	0/10	0/10	0/10	0/10
Solitary fibrous tumor	0/10	0/10	0/10	3/10	0/10	0/10
Malignant peripheral nerve sheath tumor (MPNST)	0/10	2/10	2/10	4/10	0/10	0/10
Angiosarcoma	0/10	3/10	2/10	7/10	0/10	0/10
Chondrosarcoma	0/10	0/10	0/9	8/10	0/7	0/10
Osteosarcoma (OS)	0/10	0/10	0/10	4/10	0/10	0/10
Epithelioid hemangioendothelioma	1/6	0/6	3/6	2/6	0/6	0/6
Epithelioid angiosarcoma	0/2	2/2	2/2	1/2	0/2	0/2
Biphasic synovial sarcoma	0/5	4/5	4/5	2/5	0/5	4/5
Total	3/310	55/310	87/309	66/310	11/307	60/310
Specificity	99%	82%	72%	79%	96%	81%

Intensity and proportion of staining in tumor cells were evaluated in the entire microscopic field of each specimen. Cases were defined as positive if the proportion score was more than 0. In the immunostaining of calretinin or WT-1, staining in the nucleus, but not the cytoplasm, was considered a positive sample.
