# Supplementary material for: HEG1 is a novel mucin-like membrane protein that serves as a diagnostic and therapeutic target for malignant mesothelioma
Source: Sci Rep. 2017 Mar 31;7:45768. doi: 10.1038/srep45768 (PMC5374711; doi:10.1038/srep45768)
Supplement: Supplementary Information [file srep45768-s1.pdf]

HEG1 is a novel mucin-like membrane protein that serves as a diagnostic and therapeutic target for malignant mesothelioma

Shoutaro Tsuji, Kota Washimi, Taihei Kageyama, Makiko Yamashita, Mitsuyo Yoshihara, Rieko Matsuura, Tomoyuki Yokose, Yoichi Kameda, Hiroyuki Hayashi, Takao Morohoshi, Yukio Tsuura, Toshikazu Yusa, Takashi Sato, Akira Togayachi, Hisashi Narimatsu, Toshinori Nagasaki, Kotaro Nakamoto, Yasuhiro Moriwaki, Hidemi Misawa, Kenzo Hiroshima, Yohei Miyagi, and Kohzoh Imai.

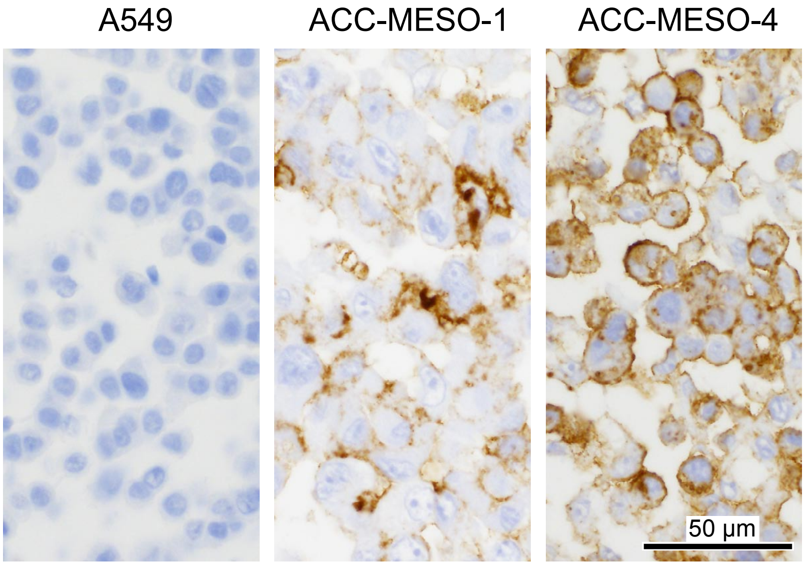

Supplementary Figure 1: Immunostaining of MPM cell lines and lung cancer cell lines using mAb SKM9-2. MPM cell lines (ACC-MESO-1 and ACC-MESO-4) and lung cancer cell lines (A549) were cultured in media containing 10% fetal bovine serum, harvested using a cell scraper, washed with phosphate-buffered saline, collected by centrifugation, and fixed by the AMeX method. The cells were prepared as paraffin-embedded thin-sliced sections and treated with mAb SKM9-2. Scale is indicated at the bottom of the image of ACC-MESO-4.

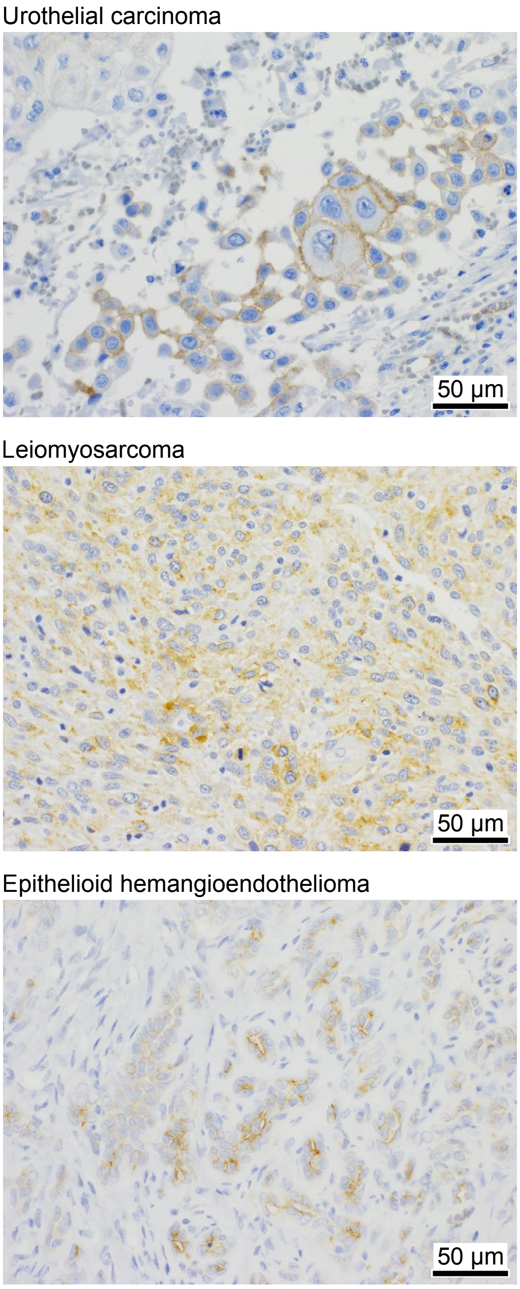

Supplementary Figure 2: Immunohistochemical staining using mAb SKM9-2 of positive cases in non-MPM tumors. Images of the three tumors judged as SKM9-2 antigen-positive are shown. Scale is indicated at the bottom of each image.

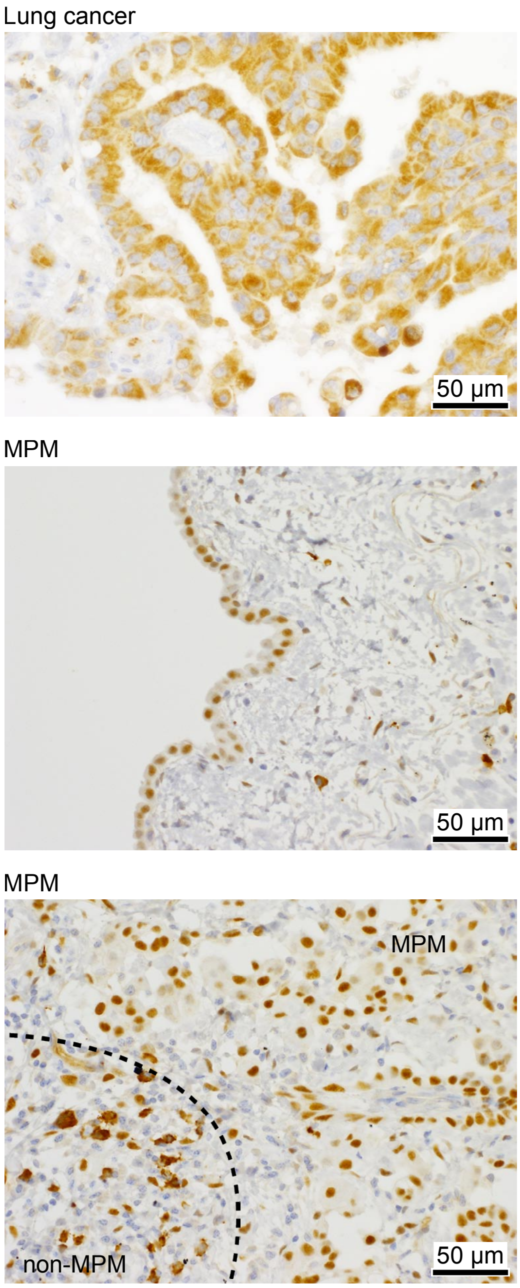

Supplementary Figure 3: Representative images of immunohistochemical staining of lung cancer or MPM using anti-WT-1 antibody. The lung cancer or MPM was stained in the cytoplasm or the nucleus, respectively. In the bottom image of an MPM case, the cytoplasmic WT-1 in non-MPM cells was stained thickly (non-MPM region in the left bottom side), whereas the nuclear WT-1 was stained in the MPM cells (MPM region). Scale is indicated at the bottom of each image.

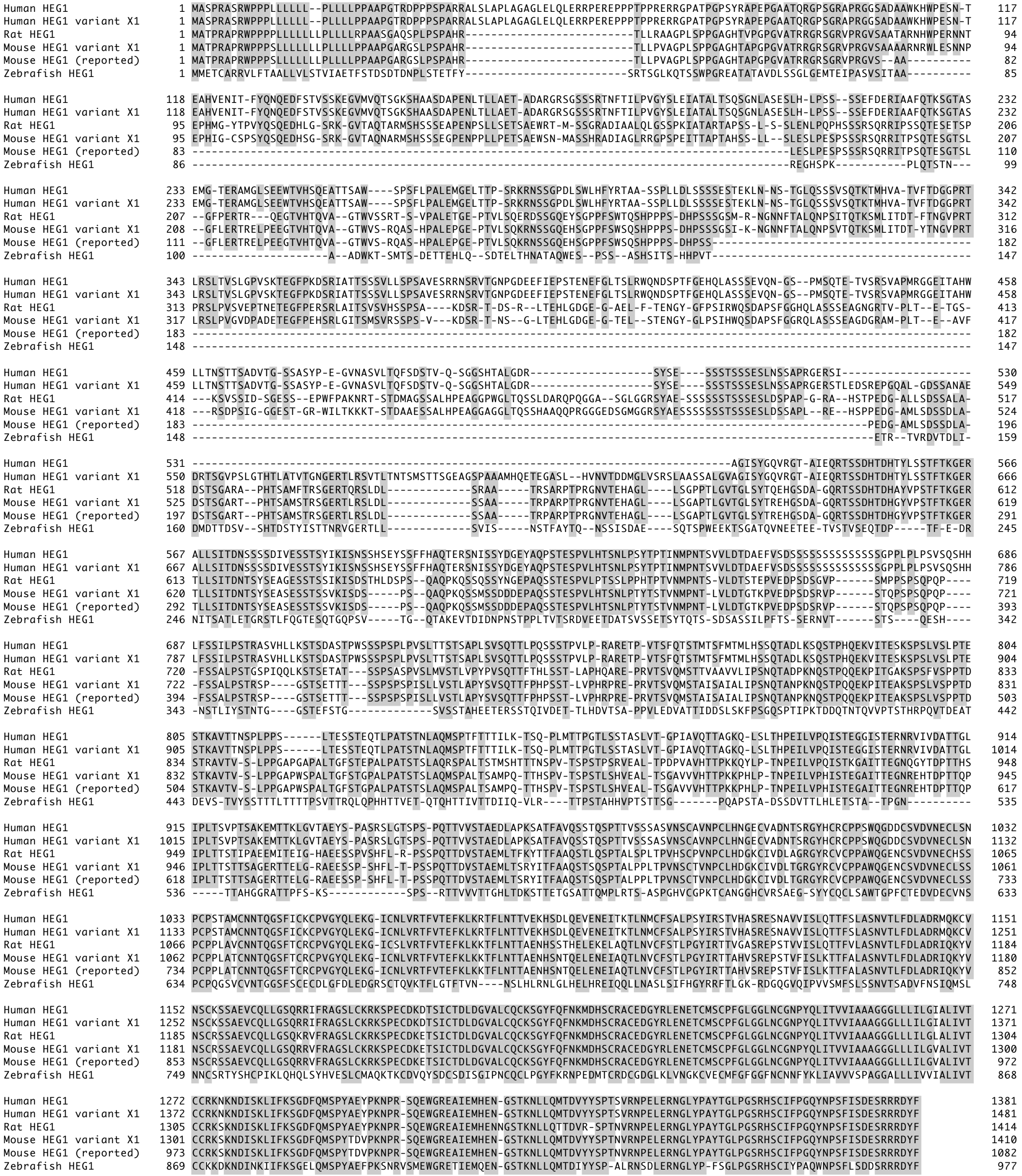

Supplementary Figure 4: Multiple alignment of homologous HEG1 amino acid sequences. Deduced amino acid sequences of HEG1 homologs are shown. Homologous amino acids are shown as shaded characters. Sequence information was obtained from GenBank database as follows: human HEG1, NM_020733; human HEG1 variant X1, XM_005247666; rat HEG1, XM_006248443; mouse HEG1 variant X1, XM_006522715; mouse HEG1 (reported), NM_175256; Zebrafish HEG1, NM_212968.

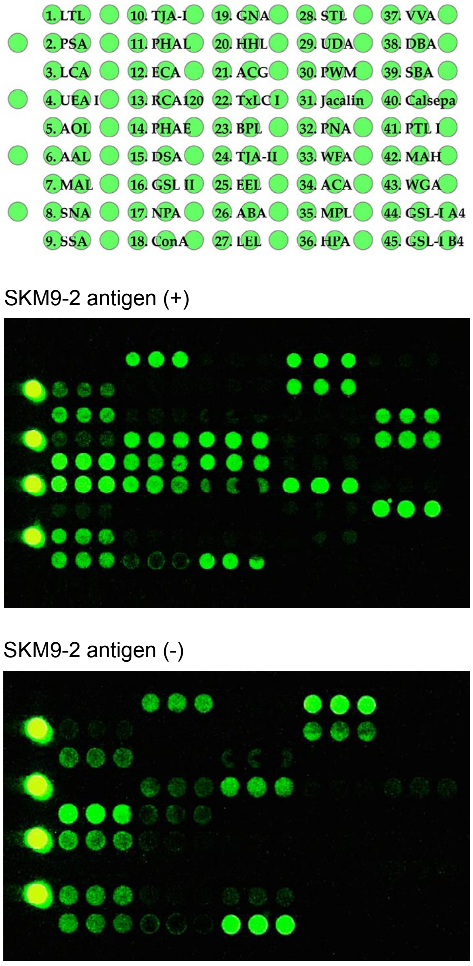

Supplementary Figure 5: Results of the lectin microarray. Treated glass slide was scanned using GlycoStation Reader 1200. Panel of spotted lectins and the results of lectin array analysis of sample with or without SKM9-2 antigen are shown.

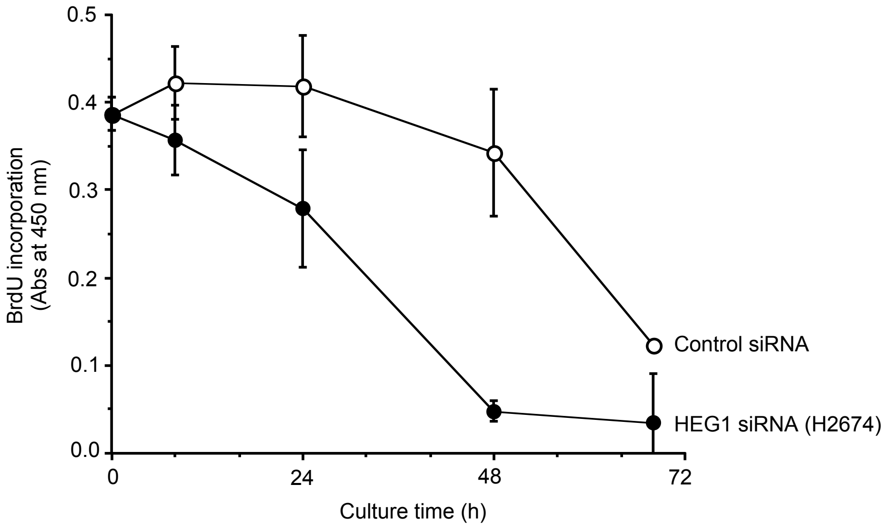

Supplementary Figure 6: Time-course of bromodeoxyuridine (BrdU) incorporation of siRNA-treated ACC-MESO-4. siRNA was transfected with Lipofectamine RNAiMAX. HEG1 siRNA, H2674; Control siRNA, MISSION siRNA Universal Negative Control (SIC-001). Values are means ± S.D. of triplicate determinations. Similar results were obtained in 2 independent experiments. The control siRNA-treated cells reached semiconfluent density after 68 h of treatment. A significant cell death of HEG1 siRNA-treated cells was observed after 48 h of treatment.

Supplementary Table 1: Expression of SKM9-2 antigen in non-tumorous tissue.

Organ		Positive No. / Sample No.	Organ		Positive No. / Sample No.
Digestive system	Tongue	0/1	Nervous system	White substance	0/13
	Esophagus	0/2		Gray substance	0/16
	Stomach	0/3		Ventricle	0/4
	Duodenum	0/2		Basal nucleus	0/2
	Small intestine	0/2		Nigra	0/2
	Colon	0/6		Cerebellum	0/1
	Appendix	0/2		Spinal cord	0/1
	Liver	0/8		Dura mater	0/1
	Pancreas	0/10		Nerve plexus	0/1
	Cholecyst	0/5	Lymphoid system	Lymph node	0/4
Urinary system	Bladder	0/4		Thymus gland	0/3
	Kidney	0/10		Spleen	0/2
	Ureter	0/1		Bone marrow	0/1
Mesothelium	Pleura	0/5	Reproductive system	Breast	0/5
	Peritoneum	0/2		Ovary	0/4
	Tunica vaginalis	0/1		Uterus	0/3
	Pericardium	1/1		Vaginal	0/1
Reactive mesothelial cells	Pleura	*3/7		Testis	1/5
Endocrine organ	Pituitary gland	0/5		Prostate	0/10
	Adrenal	0/5	Muscle tissue	Skeletal muscle	0/1
	Thyroid	0/5		Smooth muscle	0/3
	Parathyroid	0/3		Cardiac muscle	0/12
	Adipose tissue	0/5	Skin	Epidermis	0/1
Vascular	Aorta	0/3	Bone	Spondylus	0/4
	Endothelium	*5/5			
Respiratory system	Bronchus	0/12			
	Lung	0/23			
	Diaphragm	0/1			

* partially stained

Intensity and proportion of staining were evaluated in the entire microscopic field of each specimen. Cases were defined as positive if the proportion score was more than 0. The asterisk indicates the results of partially staining (10–50%). Representative images of positive cases are shown in Fig. 1c. Vascular endothelium was observed in tissue from the digestive, urinary, respiratory, and reproductive system.

Supplementary Methods

Screening of mAb against MPM. ACC-MESO-1 and ACC-MESO-4 (5 mg each) were injected with AddaVax (Invivogen, San Diego, CA, USA) into the peritoneal cavities of mice every 2 weeks. After the fourth immunization, the spleen was removed, and the immunized spleen cells were isolated. Hybridomas were cloned from PAI fused with the spleen cells using polyethylene glycol 1500 (Roche Diagnostics K.K., Tokyo, Japan) according to the manufacturer's instructions. For screening, the cell pellet of ACC-MESO-1, ACC-MESO-4, or A549 was fixed by the AMeX method (Sato, Y., Mukai, K., Watanabe, S., Goto, M. & Shimosato, Y. The AMeX method. A simplified technique of tissue processing and paraffin embedding with improved preservation of antigens for immunostaining. *Am. J. Pathol.* **125**, 431–435 (1986)), and subsequently prepared as paraffin-embedded thin-sliced sections. The section was blocked with saline containing 0.5% casein, incubated with a culture supernatant of a hybridoma, and visualized using the EnVision+ kits (Dako Japan Co., Kyoto, Japan). Several clones were isolated as hybridomas secreting mAb that bound to ACC-MESO-1, ACC-MESO-4, or some specimens of MPM, but neither A549 nor any specimens of lung adenocarcinoma. The selection of clones was proceeded as results of immunostaining to tissue samples and a clone, SKM9-2, was obtained as a hybridoma producing mAb that specifically recognized mesothelioma cells. The mAb SKM9-2 consists of mouse IgG1 heavy chain and kappa light chain. The sequences were determined using RT-PCR and the 5'-Full RACE core set (Takara Bio, Shiga, Japan).

Western blotting. Confluent monolayer cells were solubilized with 20 mM Tris buffer (pH 8.0) containing 1% SDS, 1 mM phenylmethylsulfonyl fluoride, 125 mU/mL

Benzonase Nuclease (Merck Millipore Co., Tokyo, Japan) (25 μL/ cm^2 culture surface area). The lysate was resolved by SDS-PAGE under reducing conditions and transferred to a polyvinylidene difluoride membrane (Immobilon-P; Merck Millipore Co.) by tank blotting in 10 mM CAPS (N-cyclohexyl-3-aminopropanesulfonic acid) (pH 10.5) containing 0.01% SDS. The membrane was blocked with 5% non-fat milk in 20 mM Tris buffered saline (pH 7.2) containing 0.1% Tween 20 (TBST), reacted with 1:1,000 diluted ascites of mAb SKM9-2, treated with horseradish peroxidase-conjugated goat anti-mouse IgG (Jackson ImmunoResearch, West Grove, PA, USA), and developed with Amersham ECL select (GE Healthcare UK, Buckinghamshire, UK). After detection using mAb SKM9-2, the membrane was reprobed by incubating with 20 mM Tris buffer (pH 8.0) containing 6 M guanidine HCl (pH 6.8) and 1% 2-mercaptoethanol, washed with water, reblocked with 5% non-fat milk, and treated with anti-β-actin mAb (AC-15, Sigma-Aldrich Japan K.K.).

Flow cytometric analysis. Using a cell scraper, confluent cell monolayers of ACC-MESO-4 were harvested in phosphate-buffered saline containing 0.1% BSA and 0.1% NaN_3. The harvested cells were incubated at 4 °C for 30 min with 1:1,000 diluted ascites of mAb SKM9-2 in the buffer. An irrelevant mAb (2D2, mouse IgG1) was used as a negative control. After washing, the cells were counterstained with fluorescein isothiocyanate-conjugated goat anti-mouse IgG $F(ab')_2$. The binding antibodies were analyzed on a BD Accuri C6 Flow cytometer (Nippon Becton Dickinson, Tokyo, Japan).

Deglycosylation analysis. Before SDS-PAGE, a sample was treated with Protein

Deglycosylation Mix, α2-3,6,8 Neuraminidase, O-Glycosidase, or PNGase F (New England Biolabs Japan, Tokyo, Japan), according to the manufacturer's instructions. Alternatively, a sample was treated at 37 °C for 4 h with 1 μg/mL of proteinase K. The samples were resolved by 4–15% SDS-PAGE under reducing conditions and detected by western blotting using mAb SKM9-2.

Purification of SKM9-2 antigen. ACC-MESO-4 (240×100 mm dishes) was solubilized at 4 °C with 240 mL of 50 mM Tris buffer (pH 8.0) containing 1% Triton X-100, 1% CHAPS (3-[(3-cholamidopropyl)dimethylammonio]propanesulfonate), 1 mM ethylenediaminetetraacetic acid, 50 mM NaCl, and a protease inhibitor cocktail (Complete, Roche Diagnostics K.K.). The lysate was centrifuged at 8,000×*g* for 10 min, and its supernatant was dialyzed at 4 °C for 18 h with 20 mM acetate buffer (pH 5.0). The following purification procedures were carried out at room temperature. The precipitate was collected by centrifugation at 8,000×*g* for 10 min, and SKM9-2 antigen was extracted from the precipitate with 20 mL of TBST. The extract was isolated by centrifugation at 8,000×*g* for 10 min, dialyzed for 3 h with 20 mM acetate buffer (pH 5.0), re-centrifuged at 8,000×*g* for 10 min, and buffer-exchanged with 20 mM Tris buffer (pH 8.0) containing 6 M guanidine hydrochloride using Amicon Ultra-15 100 K devices (Merck Millipore Co). The concentrated extract (1.3 mL) was reduced with 10 mM TCEP (tris(2-carboxyethyl)phosphine) at 60 °C for 30 min, alkylated with 40 mM iodoacetamide at room temperature for 3 h, and isolated by size exclusion chromatography in 20 mM Tris buffer (pH 8.0) containing 0.05% Tween 20 and 6 M guanidine hydrochloride. Chromatographic separation used a flow rate of 0.5 mL/min over a Superose 6 Increase 10/300GL column connected to an AKTAexplorer 10S (GE

Healthcare UK). The SKM9-2 antigen in the separated fractions was detected by dot blot analysis using mAb SKM9-2 (Fig. 2d). Fractions 3–10 were pooled, dialyzed at room temperature for 18 h with 25 mM phosphate buffer (pH 7.0) containing 0.05% Tween 20 and 4 M urea, and isolated by anion exchange chromatography with a Mono Q 5/50GL column (GE Healthcare UK). The antigen eluted in fractions 7–14 with a linear gradient of 0–1 M NaCl in 25 mM phosphate buffer (pH 7.0) containing 0.05% Tween 20 and 4 M urea (Fig. 2e). The pooled fraction was dialyzed with 25 mM phosphate buffered saline (pH 7.2) containing 0.05% Tween 20, applied to a WGA-agarose 1 mL column (J-Oil Mills, Tokyo, Japan), washed with the buffer, and eluted with 25 mM phosphate buffered saline (pH 7.2) containing 0.2 M N-acetylglucosamine and 0.05% Tween 20 (Fig. 2f). The eluted sample (fractions 3–11) was concentrated with an Amicon Centricon YM-30 concentrator (Merck Millipore Co.), and isolated in 25 mM phosphate buffer (pH 7.2) containing 0.5% CHAPS and 3 M guanidine hydrochloride by size exclusion chromatography (Fig. 2g) (flow rate of 0.5 mL/min; Superose 6 Increase 10/300GL column). Fractions 3–8 were pooled, concentrated, and dialyzed with 20 mM NH_4HCO_3 containing 0.5% CHAPS.

This purified sample was resolved by SDS-PAGE (4–15% Mini-PROTEAN TGX gel, Bio-Rad Laboratories, Hercules, CA, USA), and analyzed by CBB staining or western blotting using mAb SKM9-2. The CBB-stained band, which had a similar mobility to the western blot band, was cut from the gel, proteolyzed, and analyzed by mass spectrometry and Mascot search (protein identification services of Wako Pure Chemical Industries, Osaka, Japan).

Cloning of HEG1 and production of recombinant HEG1. cDNA was synthesized

from total RNA purified with Trizol reagent (Thermo Fisher Scientific, Rockford, IL, USA) from ACC-MESO-4 by using an oligo dT primer and SuperScript II RNase H reverse transcriptase (Thermo Fisher Scientific). cDNA encoding the HEG1 open reading frame was amplified by PCR using PrimeSTAR HS DNA Polymerase (Takara Bio), and the PCR products were directly sequenced using several internal sequence primers. The HEG1 cDNA was cloned and inserted into the pcDNA3.1(-) mammalian expression vector (Thermo Fisher Scientific). Two transcript variants of HEG1, with and without exon 6, were cloned. Soluble HEG1 (sHEG1), in which a His-tag was substituted for the transmembrane and cytoplasmic domains, was also prepared. The plasmid constructs were transfected into ACC-MESO-4 with Lipofectamine LTX with Plus Reagent (Thermo Fisher Scientific), or into HEK293T with Lipofectamine 3000. The transfected cells were incubated for 72 h. These cells and their culture supernatants were used for experiments.

To purify recombinant sHEG1, sHEG1 with exon6 was transfected into ACC-MESO-4 with Lipofectamine LTX with Plus Reagent. The transfected cells were selected with geneticin (Thermo Fisher Scientific), and the stably sHEG1-expressed clones were obtained. Supernatant from the clone culture (40 mL) was collected and applied to a HisTrap excel 1 mL column (GE Healthcare UK). This column was washed with 50 mM phosphate buffer containing 10 mM imidazole and 500 mM NaCl, and His-tagged protein was subsequently eluted with 50 mM phosphate buffer (pH 8.0) containing 500 mM imidazole and 500 mM NaCl. The eluate was buffer-exchanged using Amicon Ultra-15 100 K devices with 20 mM Tris buffer (pH 8.0) containing 10 mM ethylenediaminetetraacetic acid and 6 M guanidine hydrochloride, re-exchanged with 25 mM phosphate buffer (pH 7.2) containing 0.1% Tween-20, and concentrated to

200 μL. The obtained sample was used as sHEG1.

RT-PCR. Total RNA was extracted from MPM cell lines with Trizol reagent. After treating with RQ1 RNase-free DNase (Promega K.K., Tokyo, Japan), the RNA (100 ng) was amplified using a OneStep RT-PCR kit (Qiagen GmbH, Hilden, Germany). This reaction used 0.6 μM of the primers 5'-TCATCAGCACCACGTGGAGAAC-3' and 5'-TGGTAAAGGTGATGGTGAGGAAGAC-3'. The temperature program was 26 cycles of 94 °C for 30 s, 58.5 °C for 30 s, and 72 °C for 1 min. Total RNA of heart, lung, liver, colon, kidney, prostate, or testis was obtained from Takara Bio and amplified by RT-PCR without DNase treatment. PCR products were resolved by 1.5% agarose gel electrophoresis and stained with ethidium bromide. The HEG1 without exon 6 and the HEG1 variant with exon 6 were detected as 600 bp and 900 bp PCR products, respectively.

Real-time quantitative PCR. Total RNAs of organs were obtained from Takara Bio. Total RNA of ACC-MESO-4 was extracted from MPM cell lines with Trizol reagent. The first cDNA strand was synthesized from 1 μg of total RNA using PrimeScript RT Master Mix (Takara Bio). Quantitative PCR was performed in quadruplicate, in a total reaction volume of 20 μL, using Thunderbird SYBR qPCR Mix (Toyobo Co., Osaka, Japan) and 2 μL cDNA per reaction, on a 96CFX (Bio-Rad Laboratories). The PCR protocol consisted of an initial cDNA denaturation (3 min at 95 °C), followed by 40 cycles of 95 °C for 3 s and 60 °C for 30 s. Primers for amplification were as follows: HEG1, sense primer located in exon 17 (5'-TCCTCCAGATGACGGATGTG-3') and antisense primer located in exon 18 (in 5'-GACGGGTTATACTGTCCGGG-3'); human

β-actin, sense primer 5'-AGAGCTACGAGCTGCCTGAC-3' and antisense primer 5'-AGCACTGTGTTGGCGTACAG-3'. Relative mRNA levels were calculated by the -ΔΔCt method, using β-actin as an internal control.

Bromodeoxyuridine (BrdU) incorporation assay. ACC-MESO-4 was seeded in a 96-well plate (5×10^3 cells/well) and cultured for 24 h in 100 μL of culture medium. siRNA was treated as described in Methods section. After 20, 44, or 64 h, the cells were cultured for 4 h with BrdU (BrdU Cell Proliferation ELISA Kit (Abcam plc, Cambridge, UK). The incorporated BrdU was measured according to the manufacturer's instructions.
